# Change of Retinal Vessels in Different Sectors of the Parapapillary Area in Primary Open-Angle Glaucoma

**DOI:** 10.3389/fmed.2021.705829

**Published:** 2021-07-08

**Authors:** Jingyi Cheng, Hongmei Zhao, Chunhui Jiang, Xiangmei Kong, Xinghuai Sun

**Affiliations:** ^1^Department of Ophthalmology and Visual Science, Eye, Ear, Nose and Throat Hospital, Fudan University, Shanghai, China; ^2^Key Laboratory of Myopia, Ministry of Health, Fudan University, Shanghai, China; ^3^Shanghai Key Laboratory of Visual Impairment and Restoration, Fudan University, Shanghai, China; ^4^State Key Laboratory of Medical Neurobiology, Institutes of Brain Science, Fudan University, Shanghai, China

**Keywords:** retinal vessel, retinal nerve fiber layer thickness, visual field, primary open-angle glaucoma, parapapillary area

## Abstract

**Purpose:** To investigate the changes in the retinal vessels (RVs) in different sectors in patients with primary open-angle glaucoma (POAG), and their possible correlations with retinal nerve fiber layer thickness (RNFLT) and visual-field defects in the temporal parapapillary region.

**Methods:** The RV diameters, RNFLTs, and visual-field parameters were measured. The temporal parapapillary region was divided into the temporal (T, 315°-45°), temporal superior (TS, 45°-90°), and temporal inferior sectors (TI, 270°-315°). The changes in the RV diameters in each sector were determined, and their relationships with RNFLT, the mean deviation (MD), and visual field sensitivity (VFS) were examined.

**Results:** Fifty POAG patients (50 eyes) and 50 healthy subjects (50 eyes) were included. Compared with the healthy subjects, the POAG group had a significantly smaller accumulated parapapillary RV diameter (*P* < 0.001), which was positively correlated with the MD and RNFLT. When the different temporal sectors were examined, the accumulated RV diameters were significantly smaller in the POAG group than in the healthy controls in the TI and T sectors, but not in the TS sector. The accumulated diameters in the TI and T sectors were correlated with the corresponding RNFLTs (all *P* < 0.05), but only the accumulated diameter in the TI sector was correlated with the VFS.

**Conclusions:** In POAG, the changes in the RVs differed between different temporal sectors, with the most prominent changes occurring in the TI and T sectors.

## Introduction

The retinal vascular caliber is a structural marker of microvascular changes in the fundus, and studies have shown that a smaller retinal vascular caliber is correlated with thinner neuroretinal rim tissue (1), a larger vertical cup-to-disc ratio (2, 3), greater nerve fiber layer visibility (1), and the presence of glaucoma (4–6). Previous studies have examined the relationship between the retinal vessel (RV) diameter and the retinal nerve fiber layer thickness (RNFLT) in patients with primary open-angle glaucoma (POAG), but the results were contradictory (7–12). It has also been reported that the RVs in different sectors respond differently in POAG. The main manifestation of glaucomatous visual field (VF) damage is the nasal VF defect (13), including arcuate scotoma in superior or/and inferior visual field, paracentral scotomas, arcs, or nasal steps, and the main blood vessels are in the temporal region (14–16). Therefore, in this study, the RV diameters in subjects with POAG were measured by optical coherence tomography (OCT), together with the RNFLTs, VF parameters, and more attention was paid on the differences in the RVs in different sectors of the temporal parapapillary region, which was corresponding to the nasal VF.

## Materials and Methods

### Ethics Statement

This study was performed in accordance with the tenets of the Declaration of Helsinki and was approved by the Medical Ethics Committee of the Eye and ENT Hospital, Fudan University, Shanghai, China. All subjects provided written informed consent to their inclusion in the study after being provided an explanation of the study.

### Study Subjects

All subjects underwent a routine ophthalmic examination that included assessment of medical history, best-corrected visual acuity (BCVA), slit-lamp biomicroscopy, gonioscopy (only patients with glaucoma), Goldmann applanation tonometry (Haag Streit AG, Bern, Switzerland), central corneal thickness (CCT; EM-3000, Tomey, Japan), axial length (AL; IOLMaster 500, Carl Zeiss Meditech, Inc., Dublin, CA), and VF testing with Humphrey automated perimetry (SITA 24-2, Carl Zeiss Meditech, Inc., Dublin, CA). The Spectralis OCT+HRA system (Heidelberg Engineering, Heidelberg, Germany) was used to measure RNFLT and RV diameters.

### POAG Group

Patients with POAG who visited the Ophthalmology Department of the Eye and ENT Hospital, Fudan University, Shanghai, between January and June 2014 were recruited. The inclusion criteria for the POAG group were: (1) age ≥ 30 years old; (2) glaucomatous damage to the optic disc, defined as the presence of at least two of the following characteristics: cup/disc ratio ≥ 0.6, cup/disc asymmetry > 0.2, diffuse or focal neural rim thinning, disc hemorrhage, localized rim loss, or nerve fiber layer defects indicative of glaucoma; (3) typical and reliable VF defects, defined as the presence of at least two of the following: a cluster of ≥3 non-edge points detected with Humphrey automated perimetry, all of which were depressed on the pattern deviation plot at the *P* < 5% level and one of which was depressed at the *P* < 1% level, or a glaucoma hemifield test result outside the normal limits, together with a pattern standard deviation of <5% of normal fields, fixation losses of ≤20%; or ≤15% false-positive or false-negative responses; and (4) a normal anterior chamber and open angle based on slit-lamp and gonioscopic examinations.

Patients were excluded if one or more of the following was present: (1) any other ophthalmic disease, such as trauma, inflammation, or retinal or neurological problems that could result in optic-nerve or VF defects; (2) patients with a shallow anterior chamber, narrow angle, or angle closure based on slit-lamp and gonioscopic examinations; (3) diopters < −6 D or > +1 D; or (4) AL > 26 mm or <20 mm; (5) smokers, or those with excessive alcohol consumption, hypertension, diabetes mellitus, or any other circulatory system disease; and (6) patients deemed unsuitable for the study by the researchers.

### Control Group

Healthy, non-smoking volunteers were recruited. The inclusion criteria were: (1) BCVA > 0.8; (2) diopters between +1 and −6 D; (3) intraocular pressure (IOP) ≤ 21 mmHg; and (4) AL of 20–26 mm. Individuals with an ocular disease, excessive alcohol consumption, hypertension, diabetes mellitus, any other circulatory system disease, or those with VF test results outside the normal limits were excluded.

### Main Ophthalmic Measurements and Analysis

**Figure 1:**
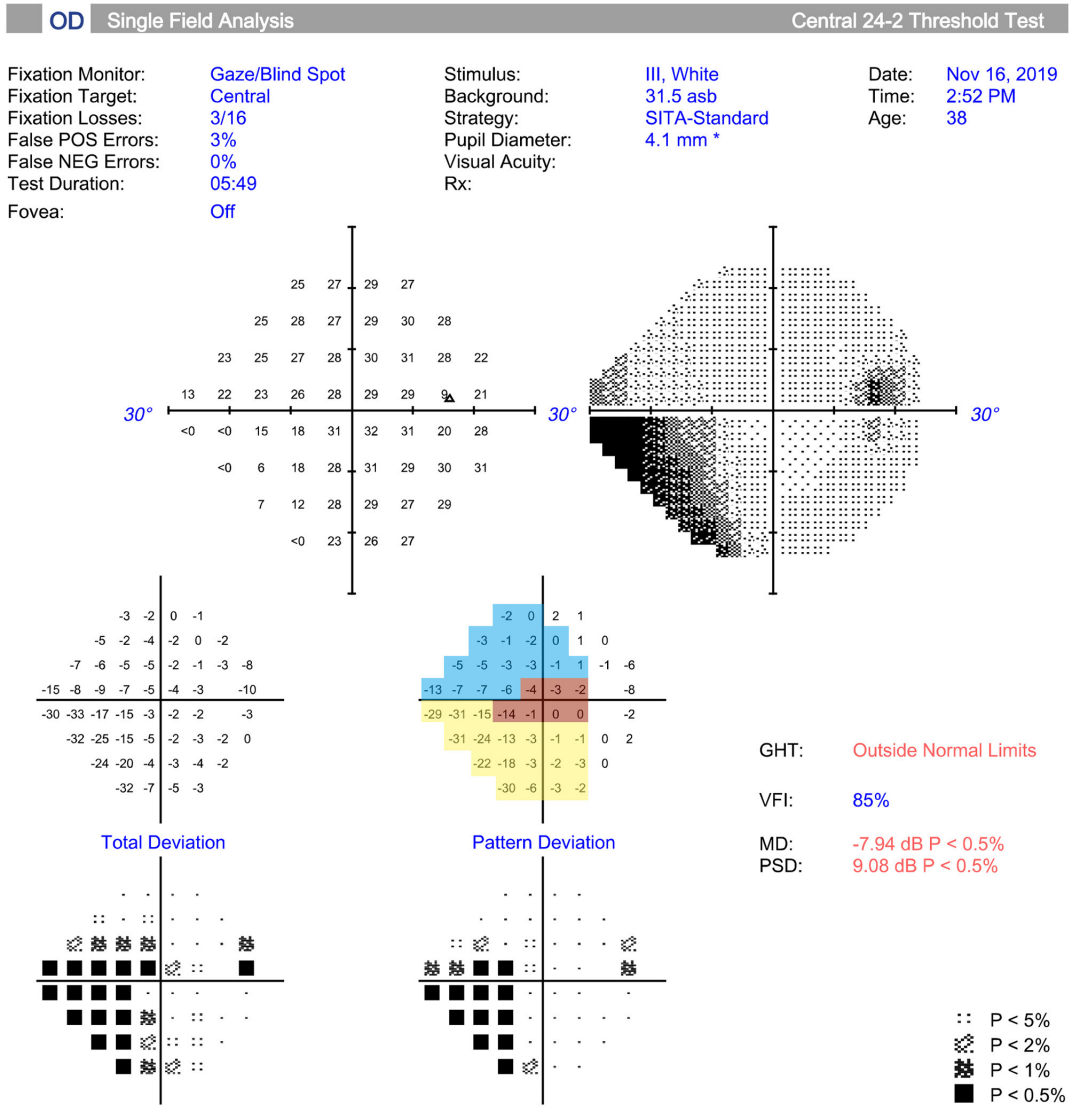
Measurement and regionalized of the visual field (VF) were demonstrated. The VF was tested (Humphrey automated perimetry SITA 24-2), and regionalized into the temporal (T) (315°-45°, red area), temporal superior (TS) (45°-90°, yellow area), and temporal inferior (TI) (270°-315°, blue area) sectors according to previous study. Regional visual field sensitivity (VFS) was calculated as the sum of the pattern deviations in a specific sector.

The VF was tested and different sectors were divided by the method described by Kanamori and Akiyasu (17). Among those sectors, three sectors were evaluated: temporal (T; 315°-45°), temporal superior (TS; 45°-90°), and temporal inferior (TI; 270°-315°) sectors (Figure 1). The regional VF sensitivity (VFS) was calculated as the sum of the pattern deviations in the corresponding sector.

**Figure 2:**
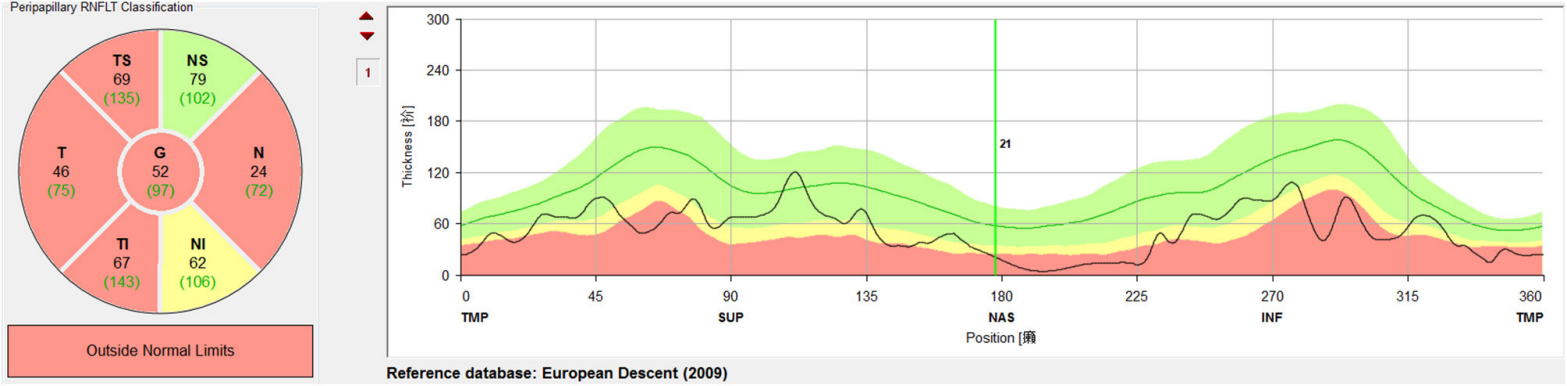
Measurement and regionalized of the retinal nerve fiber layer thickness (RNFLT) were demonstrated. A circular scan with a 3.4 mm diameter centered on the optic nerve head was performed through undilated pupils with the Spectralis OCT + HRA system. The average parapapillary RNFLT(G) and the average RNFLTs in the T (315°-45°), TS (45°-90°), and TI (270°-315°) sectors were obtained with the Spectralis viewing software (Heidelberg Eye Explorer, version 1.0.10.0).

A circular scan with a 3.4 mm diameter centered on the optic nerve head was performed through undilated pupils with the Spectralis OCT + HRA system. The automatic real time was set to 100 for maximal quality and resolution of the OCT images. Near-infrared reflectance (IR) images were obtained simultaneously. The average parapapillary RNFLT and the average RNFLTs in the T (315°-45°), TS (45°-90°), and TI (270°-315°) sectors were determined using the Spectralis software (Heidelberg Eye Explorer, version 1.0.10.0) (Figure 2).

**Figure 3:**
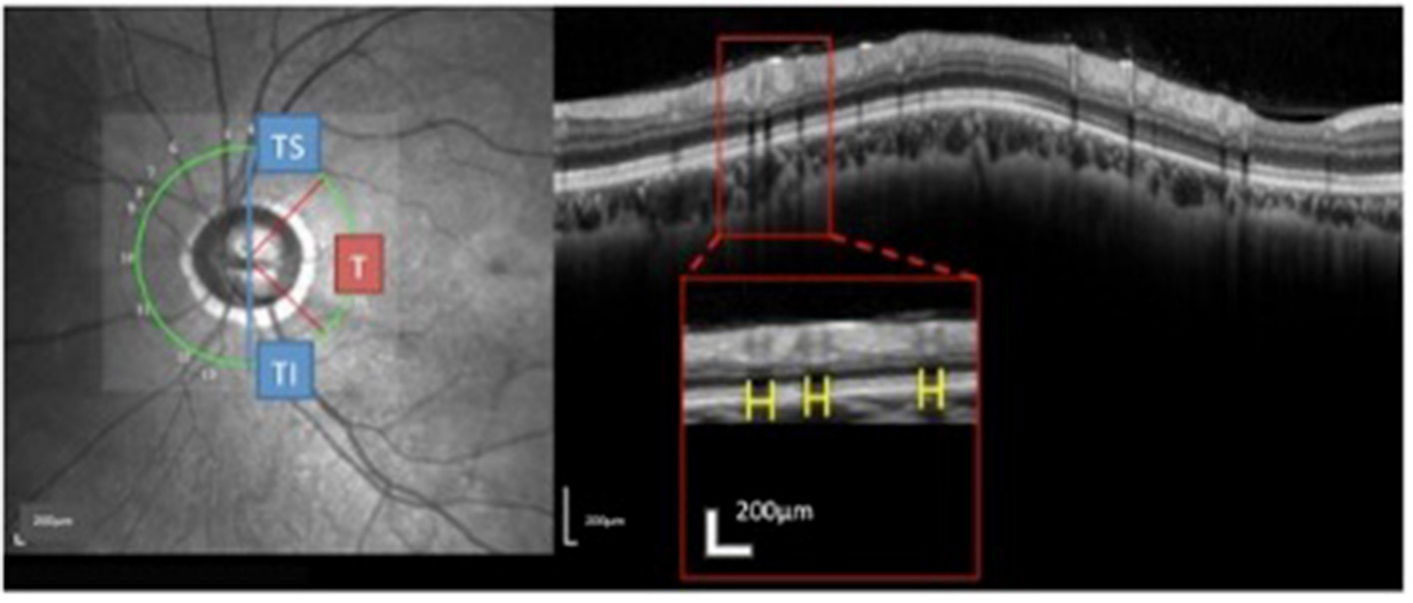
Measurement and regionalized of the retinal vessels (RV) diameter were demonstrated. Using the Spectralis OCT + HRA system, a circular scan with a 3.4 mm diameter centered on the optic nerve was performed through natural pupils. Near-infrared reflectance images were obtained simultaneously. The retinal vessel (RV) diameter was measured as the maximum reflectance shadowing width on the retinal pigment epithelium layer. Using Spectralis software (Heidelberg Eye Explorer, version 1.0.10.0), scales (yellow bar) were drawn and measured. The sums of the overall parapapillary RV diameters, and the average parapapillary RNFLTs were determined for the entire region and for the T (315°-45°), TS (45°-90°), and TI sectors (270°-315°) individually.

The RVs were identified on the OCT images based on the near-infrared images. The method used to measure the RV diameters on spectral domain OCT has been described (18). Briefly, the brightness and contrast settings used for the IR images were: “black on white” for the color table, “medium” for sharpening, and “none” for noise reduction. The RV diameter was measured as the maximum reflectance shadowing width on the retinal pigment epithelium (RPE) layer. The RV diameter obtained from IR images was measured vertically to the vessel axis with the caliber tool from the Spectralis software at the crossing point of vessel and OCT scanning line. The overall sums of the parapapillary RV diameters and the sums for the T (315°-45°), TS (45°-90°), and TI (270°-315°) sectors were calculated (Figure 3) and listed as the accumulated RV diameters of the parapapillary and different sectors.

### Data and Statistical Analysis

One eye from each of the eligible POAG subjects and the healthy subjects was randomly selected for the study using a web-based research randomization tool (19). Statistical analyses were performed using SPSS for Mac, version 23.0. Categorical variables were described as frequencies and constituent ratios, and were analyzed with χ^2^ tests. Continuous variables were described as means and standard deviations (SD). Statistical comparisons between the POAG patients and healthy subjects were made using independent-samples two-tailed *t-*tests. The correlations between the accumulated parapapillary RV diameter and RNFLT or VFS were determined using Pearson's correlation coefficient. A *P*-value < 0.05 was defined as statistically significant.

## Results

### Demographic and Ophthalmic Characteristics of the Participants

In total, 50 POAG patients (50 eyes) and 50 healthy subjects (50 eyes) were included in the study. The POAG group had a higher IOP than the control group (*P* < 0.001) and all of the enrolled POAG patients used only one antiglaucoma medication, a prostaglandin analog. The sex and age distributions and other characteristics of both groups were similar (Table 1).

**Table 1:** Demographics and ophthalmic characteristics of the POAG and control groups.

	**POAG group *N* = 50**	**Control group *N* = 50**	***t***	***P*-value**
Age	43.50 ± 13.36	38.92 ± 10.75	1.889	0.06
Sex (*n*, %)			2.966	0.23
Male (*n*, %)	28 (56%)	28 (56%)		
Female (*n*, %)	22 (44%)	22 (44%)		1.00
IOP (mmHg)	22.03 ± 5.75	14.76 ± 4.97	5.537	<0.001^*^
BCVA	0.88 ± 0.21	0.96 ± 0.22	−0.773	0.45
Diopters	−3.54 ± 2.52	−2.46 ± 3.00	−0.977	0.34
CCT (μm)	527.17 ± 42.53	540.92 ± 37.89	0.188	0.09
AL (mm)	24.67 ± 1.22	24.22 ± 1.18	1.895	0.06
MD	−8.27 ± 4.99	−1.44 ± 1.14	−5.053	<0.001^*^
PSD	8.44 ± 4.31	1.58 ± 0.29	5.932	<0.001^*^

*Statistical comparisons between the POAG and control groups were made with a χ^2^ test or an independent-samples two-tailed t-test*.

*POAG, primary open-angle glaucoma; IOP, intraocular pressure; BCVA, best corrected visual acuity; CCT, central corneal thickness; AL, axial length; MD, mean deviation; PSD, pattern standard deviation*.

* *Significantly different between the POAG and control groups at P < 0.05*.

### Accumulated RVs and RNFLTs in the POAG Group

The POAG group had significantly smaller accumulated parapapillary RV diameters and thinner parapapillary RNFLTs (*P* < 0.001) than the control group (Table 2). For the individual temporal sectors, the RNFLTs in the T, TS, and TI sectors were significantly thinner (all *P* < 0.001), and the accumulated RV diameters in the T and TI sectors were significantly smaller (*P* = 0.001 and *P* = 0.002, respectively) in the POAG group than the control group.

**Table 2:** Comparisons of RVs and RNFLT between the POAG and control groups.

	**POAG group *N* = 50**	**Control group *N* = 50**	***t***	***P*-value**
**Vessel diameter**				
Parapapillary	1096.08 ± 158.62	1280.08 ± 156.01	−5.848	<0.001^**^
TS	247.58 ± 75.35	262.50 ± 71.25	−1.017	0.31
TI	217.64 ± 59.21	264.12 ± 71.88	−3.529	0.001^**^
T	126.28 ± 68.31	173.70 ± 83.12	−3.117	0.002^*^
**RNFLT**				
Parapapillary	65.46 ± 14.42	101.26 ± 13.32	−12.897	<0.001^**^
TS	91.76 ± 30.84	140.66 ± 21.20	−9.239	<0.001^**^
TI	63.72 ± 30.25	158.68 ± 28.54	−16.147	<0.001^**^
T	63.54 ± 20.11	88.24 ± 17.34	−6.578	<0.001^**^

*Continuous variables are given as means ± SD. Statistical comparisons of the POAG and control groups were made with an independent-samples two-tailed t-test*.

*RV, retinal vessel; RNFLT, retinal nerve fiber thickness; POAG, primary open-angle glaucoma; T, temporal; TS, temporal superior; TI, temporal inferior*.

* *Significantly different between the POAG and control groups at P < 0.05*.

** *Significantly different between the POAG and control groups at P < 0.001*.

### Correlations Among Accumulated RVs, RNFLT, and VFS

In the POAG group, the accumulated parapapillary RV diameter was positively correlated with the MD value (r = 0.30, *P* = 0.02) and the RNFLT (r = 0.40, *P* = 0.004). For the individual sectors, the accumulated RV diameter in the TI sector was correlated with the corresponding VFS (r = 0.28, *P* = 0.03) and RNFLT (r = 0.45, *P* = 0.001). The accumulated RV diameter in the T sector was correlated with the corresponding RNFLT (r = 0.49, *P* < 0.001) but not with VFS (r = 0.18, *P* = 0.15). No significant correlations were detected in the TS sector (Table 3).

**Table 3:** Correlations between RV diameters and VFS or RNFLT within the parapapillary region and individual temporal sectors.

		**Parapapillary**	**Temporal**	**Temporal superior**	**Temporal Inferior**
VFS	*r*	0.30	0.18	−0.04	0.28
	*P*-value	0.02^*^	0.15	0.77	0.03^*^
RNFLT	*r*	0.40	0.49	0.27	0.45
	*P*-value	0.004^*^	<0.001^**^	0.06	0.001^*^

*Pearson's correlation coefficient was used to determine correlations between the regional/sector RV diameters and VFS or RNFLT*.

*RV, retinal vessel; RNFLT, retinal nerve fiber layer thickness; VFS, visual-field sensitivity*.

* *Significant correlation at P < 0.05*.

** *Significant correlation at P < 0.001*.

## Discussion

To analyze the differences in the change or damage of RVs in the parapapillary areas in POAG eyes, especially in the temporal side, a group of patients with POAG and healthy controls were recruited. The accumulated parapapillary RV diameter was significantly smaller in the POAG group, and the reductions were most prominent in the TI and T sectors in the studied area. The accumulated parapapillary RV diameter was correlated with both RNFLT and the MD, but when the sectors were analyzed individually, the accumulated RV diameter was only significantly correlated with both RNFLT and VFS in the TI sector.

Consistent with previous studies that analyzed the changes in RV in glaucomatous eyes (6, 20, 21), this study shows that the accumulated parapapillary RV diameter was significantly smaller in the POAG group than in the control group. More importantly, we found that, whereas RNFLT was significantly reduced in all three temporal sectors, the RV diameter was significantly reduced in the TI (*P* = 0.001) and T (*P* = 0.002) sectors, but not in the TS sector (*P* = 0.31) (Table 2). Previously, Jonas et al. (7) reported that the RV diameter was significantly smaller in glaucomatous eyes, and that the difference was greatest for the TI artery. The more-severe damage to the vessels in the TI sector is consistent with the clinical observation of more frequent and more-severe damage to the neuroretinal rims in TI (22, 23) and the more-pronounced VF changes in the upper nasal quadrant (8, 13). The reason for this is not fully understood, but might be explained as follows. In healthy subjects, Shahidi et al. (24) found that the retinal oxygen saturation was significantly greater in the TS arterioles and venules than in the TI vessels. Moreover, the TI sector of the parapapillary retina was less responsive to vasodilation and more responsive to vasoconstriction than the TS sector (25). When Gazzard and Gus (13) compared the characteristics of the VF defects between patients with primary angle-closure glaucoma (PACG) and patients with POAG, they found that POAG occurs more frequently in the superior hemifield compared with PACG. The authors speculated that under certain conditions, such as glaucoma, the responses of the vessels in the TS and TI quadrants differ and contributes to the different susceptibilities of VF defects or vascular dysfunction in these sectors. This might indeed reflect non-pressure-related optic neuropathy. In this study, the RVs in the TI sector were most frequently impaired in the POAG eyes. Because IOP should be the same throughout the eye, and IOPs of some patients were in the normal range, the more-damaged vessels in the TI sector support the non-pressure-related reason in another way. Therefore, the RVs in different sectors may differ or be affected differently under different pathological conditions, so it is important to compare vessels within the same region.

The accumulated parapapillary RV diameter was correlated positively with MD and RNFLT, but when the sectors were examined individually, only the accumulated RV diameter in the TI sector was correlated with both the corresponding RNFLT and VFS. The relationship between the glaucomatous structural and functional changes is complex and the relationship between the reduced retinal blood flow and glaucoma remains controversial (7–12). For example, using fundus photographs, Zheng et al. (10) found a significant association between a narrower RV caliber and RNFL thinning in the healthy population, but not in patients with glaucoma. Although blood vessels themselves account for ~9% of the total RNFL area, studies had found that blood vessel locations outside the optic nerve head are sufficiently stable over glaucoma severity to represent individual eye anatomy and confirmed close relationship of blood vessel locations and RNFL thickness since arteries having lower diameters (14, 16). Consistent with our results, Jonas et al. (7) showed that the retinal arteries were significantly wider where the rim area was larger, the rim notch was more distinct, and the RNFL score was higher. Radcliffe et al. (15) found a retinal blood vessel shifts in those eyes with functionally progressive glaucoma, as a result of adjacent neural tissue damage. Hall et al. (8) found a strong association between a reduced peripapillary arteriole diameter and VF defects in the corresponding hemifield in eyes with POAG. This also indicates a spatial correlation between a reduction in the vessel diameter and the glaucomatous damage in the corresponding region. The discrepancies in these findings may be attributable to the different instruments or analytical methods used, the different populations, or the severity of the disease.

It is interesting to note that the accumulated RV diameter in the T sector was correlated with the corresponding RNFLT, but not with the corresponding VFS. Many studies have presented evidence that structural damage often precedes detectable functional changes, and that the association between structure and function increases with increasing disease severity (26). It has been reported that VF abnormalities do not develop until a certain number (at least 25–35%) of retinal ganglion cells are lost (26, 27). The T sector includes the macular area, and has more ganglion cells than the other sectors. It was speculated that the neighboring retinal ganglion cells compensate for neighboring dead or dying cells until most of the cells in a region are no longer functional (26). This might partly explain our findings.

This study was limited by the relatively small sample and its cross-sectional design. Further research with more patients is required. A prospective study is still necessary to investigate the relationship between the vascular structure and the functional damage in glaucoma patients.

In conclusion, the RVs in different parapapillary regions change differently in POAG patients, and the vessels in the TI and T sectors seem to be most susceptible to damage.

## Data Availability Statement

The raw data supporting the conclusions of this article will be made available by the authors, without undue reservation.

## Ethics Statement

The studies involving human participants were reviewed and approved by the Medical Ethics Committee of the Eye and ENT Hospital, Fudan University, Shanghai, China. The patients/participants provided their written informed consent to participate in this study.

## Author Contributions

JC collected data, performed analyses, and wrote the manuscript. HZ collected data and revised the manuscript. CJ revised the manuscript, gained the fund, and supervised the process. XK provided patients' information. XS gained the fund and supervised the process. All authors contributed to the article and approved the submitted version.

## Conflict of Interest

The authors declare that the research was conducted in the absence of any commercial or financial relationships that could be construed as a potential conflict of interest.
